# A General Framework for Formal Tests of Interaction after Exhaustive Search Methods with Applications to MDR and MDR-PDT

**DOI:** 10.1371/journal.pone.0009363

**Published:** 2010-02-23

**Authors:** Todd L. Edwards, Stephen D. Turner, Eric S. Torstenson, Scott M. Dudek, Eden R. Martin, Marylyn D. Ritchie

**Affiliations:** 1 Division of Epidemiology, Department of Medicine, Vanderbilt Epidemiology Center, Vanderbilt University School of Medicine, Nashville, Tennessee, United States of America; 2 Center for Human Genetics Research, Vanderbilt University Medical Center, Nashville, Tennessee, United States of America; 3 Center for Genetic Epidemiology and Statistical Genetics, John P. Hussman Institute for Human Genomics, University of Miami Miller School of Medicine, Miami, Florida, United States of America; Institute of Preventive Medicine, Denmark

## Abstract

The initial presentation of multifactor dimensionality reduction (MDR) featured cross-validation to mitigate over-fitting, computationally efficient searches of the epistatic model space, and variable construction with constructive induction to alleviate the curse of dimensionality. However, the method was unable to differentiate association signals arising from true interactions from those due to independent main effects at individual loci. This issue leads to problems in inference and interpretability for the results from MDR and the family-based compliment the MDR-pedigree disequilibrium test (PDT). A suggestion from previous work was to fit regression models *post hoc* to specifically evaluate the null hypothesis of no interaction for MDR or MDR-PDT models. We demonstrate with simulation that fitting a regression model on the same data as that analyzed by MDR or MDR-PDT is not a valid test of interaction. This is likely to be true for any other procedure that searches for models, and then performs an uncorrected test for interaction. We also show with simulation that when strong main effects are present and the null hypothesis of no interaction is true, that MDR and MDR-PDT reject at far greater than the nominal rate. We also provide a valid regression-based permutation test procedure that specifically tests the null hypothesis of no interaction, and does not reject the null when only main effects are present. The regression-based permutation test implemented here conducts a valid test of interaction after a search for multilocus models, and can be applied to any method that conducts a search to find a multilocus model representing an interaction.

## Introduction

Methods to detect epistasis, or gene-gene interactions, in large search spaces have been under development from several sources since Risch et al. suggested interaction analysis as an important avenue for the discovery of genetic exposures related to complex disease [1]. Multifactor dimensionality reduction (MDR) has been a popular approach to search for gene-gene interactions [2]–[7]. MDR and it's family-based compliment, the MDR pedigree disequilibrium test (MDR-PDT) [8] are nonparametric methods that do not require the specification of a genetic model profiling risk or the estimation of any population parameters. MDR has good power to detect purely epistatic effects in simulated data under a variety of circumstances [7], [9]–[11]. One reason for the efficiency of MDR is the use of constructive induction [12] to develop the trait model. As a result, MDR and MDR-PDT both mitigate the curse of dimensionality [13], are sensitive to variation in penetrance across genotypes, and are not subject to model-building constraints, such as those imposed in methods that condition on marginal effects to restrict search spaces [3]. Another reason for this efficiency is the permutation test procedure that MDR uses to estimate the significance of a result; which exactly accounts for locus non-independence among loci due to linkage disequilibrium and test statistic correlation among multilocus interaction models which share loci, such as the models (SNP1×SNP2) and (SNP1×SNP3). This occurs because correlations in the data are naturally represented as correlated test statistics in the estimate of the distribution of the null hypothesis, providing the correct critical value for the test statistic even in the presence of complex data correlations.

However, the MDR and MDR-PDT approaches share some deficiencies. In order to maximize the flexibility with which models are assessed, no formal test of interaction is performed. This means that while MDR is sensitive to detect multi-locus association signals; these associations can be due to true interactions associating with the trait or multiple main effects without interaction. Both situations will lead to rejection of the null hypothesis of no association between genotypes and disease from the permutation test. Since association of multi-locus genotypes with the trait is the formal alternative hypothesis of the MDR and MDR-PDT hypothesis tests, this is not technically a type I error; however, if one is primarily interested in detecting true interactions and not simply finding combinations of associated loci, a rejection of the null ought to reliably represent support for the alternative hypothesis of interaction. With the original structure of the hypothesis test, this issue remained ambiguous after hypothesis testing, and obtaining an unbiased evaluation of MDR or MDR-PDT model properties with regard to synergy required an independent sample.

In contrast, parametric statistics such as logistic regression [14] have limited utility when searching for interactive effects in a large search space, whether searching through genetic loci [15] or environmental exposures [16]. These methods do not natively adjust for many comparisons or accommodate scenarios with high dimensionality. As the number of predictor variables increases, the number of comparisons necessary to explore the entire epistatic search space expands rapidly, decreasing the power to reject the null after an inefficient correction for multiple tests. However, these methods do provide some important advantages over the nonparametric alternatives, such as estimation of population parameters, adjustment for covariates, and ease of use and interpretability. A further advantage of the regression framework is the specificity of the hypothesis tests, particularly for testing interaction. While MDR and MDR-PDT test one composite null hypothesis, H_0A_: no association (main effects) and H_0I_: no interaction, regression is able to evaluate the null hypothesis in two parts, allowing a test for H_0I_ versus H_1I_. This property of regression-based hypothesis testing is necessary for an algorithm to find reliable evidence for epistasis in genetic studies.

It has been proposed to apply stepwise logistic regression and include only factors that exhibit a significant main effect in the final model [17]; however, interactive effects among SNPs with statistically undetectable or weak main effects are not likely to be detected, and higher order interaction models have many degrees of freedom and sparse observations. For the purpose of detecting two-locus models, an exhaustive regression-based approach with a conventional correction for multiple tests is more powerful than multi-stage regression conditioning on significant main effects [18]. However, computation times for exhaustive searches with iterative methods for fitting regression models can be very long. Methods that are optimized for computational speed that also correct for multiple tests appropriately given linkage disequilibrium and non-independent multilocus models under consideration are needed to effectively search the genome for gene-gene interactions. Advances in this regard are ongoing, with the implementation of extreme value distributions to increase the speed of permutation testing by 50-fold [19]. Other computational optimizations currently in development are the use of parallel computing [20] and hardware acceleration using graphics processing units [21] to increase computation speed. MDR and MDR-PDT handle several of these important issues, such as dimensionality, multiple comparisons, and over-fitting using cross-validation [22], [23]; and regression effectively handles some other issues, such as specificity of hypothesis tests, interpretability, and effect size estimation.

It is intuitive that evaluation of a large number of models for nonrandom association of multi-locus genotypes with a trait when looking for interactions could cause bias. Here we examine the extent of this bias with simulation and propose an alternative means of testing the null hypothesis of no interaction using a regression framework.

## Results

### Type I Error of Regression after MDR or MDR-PDT

The Type I error rate of the LRT for the regression interaction term corresponding to MDR-PDT two-locus models when compared to the chi-squared, one degree of freedom distribution was 0.39 at an alpha rate of 0.05. For logistic regression tests of interaction in case-control data on the best model from MDR analysis the type I error rate was 0.46 at an alpha rate of 0.05.

The Type I error rate for the experimental scenario where random pairs of loci were chosen, genotypes given a binary coding using constructive induction, followed by calculating a LRT statistic for the interaction term was 0.048 in pedigree data and 0.047 in case-control data at an alpha rate of 0.05.

### Type I Error of the LRT

For MDR-PDT the regression-based permutation test was conducted in 1000 500-DSP datasets with no penetrance function and 1000 permutations. The type I error rate of the test was 0.011 at an alpha rate of 0.01, 0.056 at an alpha rate of 0.05, and 0.097 at alpha 0.1.

For MDR the regression-based permutation test was conducted in 1000 datasets with 500 cases and controls with no penetrance function and 1000 datasets. The type I error rate of the test was 0.009 at an alpha rate of 0.01, 0.049 at an alpha rate of 0.05, and 0.082 at an alpha rate of 0.1.

### MDR and MDR-PDT in the Presence of Independent Main Effects

Both MDR and MDR-PDT reject the null hypothesis for groups of non-interacting associated loci at higher than the nominal rate (Table 1). This behavior is more acute as sample and effect sizes increase. The rate at which the LRT in the absence of effect modification finds the main effect model and rejects the null was zero, demonstrating that the specificity for interactions, defined as 1-(false positive rate), is superior to that of the conventional permutation test.

**Table 1:** Rejection of the null hypothesis in MDR-type algorithms when only main effects are present is more evident as sample and effect sizes increase.

	500 families		2000 families	
Relative Risk	MDR-PDT	MDR-PDT LR	MDR-PDT	MDR-PDT LR
1.5	0	0	0	0
2	0	0	1	0
4	2	0	24	0
6	4	0	50	0

	500 cases/controls		2000 cases/controls	
Relative Risk	MDR	MDR-LR	MDR	MDR-LR
1.5	10	0	60	0
2	42	0	73	0
4	67	0	83	0
6	83	1	92	0

This behavior is not observed in the LR test, where one significant result was observed in 800 replicates, compared with 591 total significant results for MDR and MDR-PDT over all parameters.

### Power of the LRT Permutation Test

**Figure 1:**
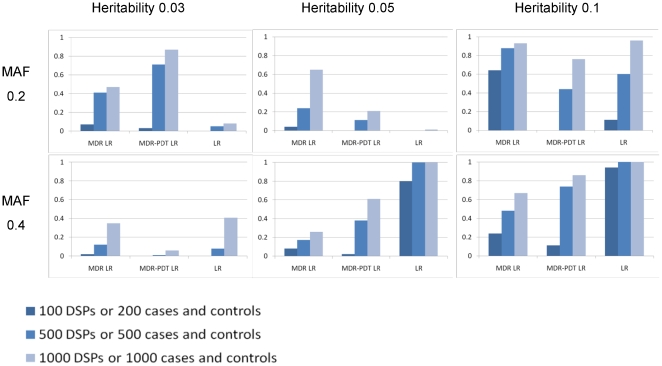
Power of different approaches for 2-locus models. This figure shows the power of MDR LR, MDR-PDT LR, and exhaustive logistic regression (LR) with a Bonferroni correction for 1225 2-locus models under six 2-locus purely epistatic genetic scenarios (Table 1) and three sample sizes for LR, MDR LR, and MDR-PDT LR. LR and MDR LR simulations were with 50 SNPs and MDR-PDT LR simulations were with 20 SNPs.

**Figure 2:**
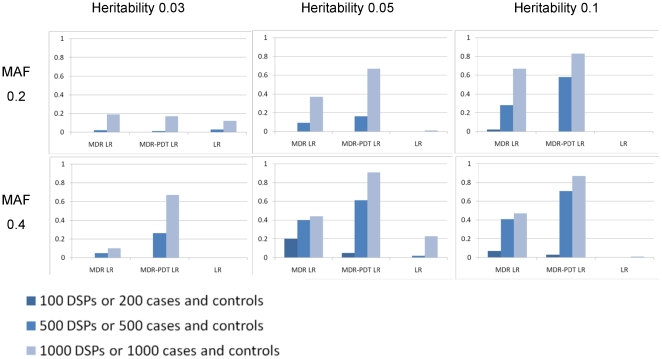
Power of different approaches for 3-locus models. Power of MDR LR, MDR-PDT LR, and exhaustive logistic regression (LR) with a Bonferroni correction for 20,825 2 and 3-locus models under six 3-locus purely epistatic genetic scenarios (Table 1) and three sample sizes for LR, MDR LR, and MDR-PDT LR. LR and MDR LR simulations were with 50 SNPs and MDR-PDT LR simulations were with 20 SNPs.

These experiments investigate the power of permutation testing of the MDR and MDR-PDT procedures using the regression-based permutation tests (MDR LR and MDR-PDT LR). The results from those experiments are presented in Figures 1–2. These results show that the regression-based permutation is notable for having power in multilocus models displaying no marginal main effect. The power of MDR LR and MDR-PDT LR are for unrelated vs. related samples, and in different numbers of markers. This was done because MDR is more powerful per sample than MDR-PDT and increasing the size of the search space provided observations for MDR that were not near 100% power. Also of note was the comparable power of the MDR-based procedures with the exhaustive search using logistic regression in cases and controls followed by a Bonferroni correction for multiple tests for most 2-locus models, and the superior performance for all 3-locus models.

## Discussion

We have introduced an extension to both MDR and MDR-PDT: a test of effect modification that has much higher specificity than the previous method of hypothesis testing. When strong main effects are present in a dataset, MDR or MDR-PDT might find a model, test, and reject the null hypothesis for these loci. This was observed in an analysis of late-onset Alzheimer's disease, when all possible 2 and 3-locus MDR and MDR-PDT models that included APOE were significant according to the permutation test [24]. However, the regression analysis of those models for interactions did not reveal any models that survived a correction for multiple tests. This occurred due to the strong main effect of APOE in the data, and the alternative hypothesis of nonrandom association for MDR and MDR-PDT, rather than interaction. Without the LRT permutation test, if the null is rejected, these findings could lead to incorrect inferences with regard to the null hypothesis of no interaction. Failure to replicate results has led some to propose that rigorous validation criteria be applied to MDR models and doubt their validity [25]. More than 100 published articles feature MDR applications or methodological extensions. A listing of published analyses featuring MDR can be found at: (http://compgen.blogspot.com/2006/05/mdr-applications.html). Because this method is in widespread use in many studies, improvements to the algorithm that increase the specificity of the hypothesis test should improve the chances of replicable findings in a large number of investigations.

It is more likely in real data that a result that rejects the more specific null hypothesis of no interaction from the regression-based test will replicate as an interaction in an independent sample than a result that rejects the general null of no association. Therefore, we advocate tests that provide high specificity and interpretability, even at the expense of some statistical power. This extension can be used as a second step in an analysis to verify interaction, conditional on observing a significant MDR or MDR-PDT result.

We also investigated the effect on type I error introduced in parametric statistics when tests are performed for MDR or MDR-PDT models in the same data where the models were found. We found that such procedures do not control the type I error rate well. The reason for this is the many comparisons which were performed to find the best model. When the threshold for significance is not corrected for this search, then the type I error rate becomes larger than expected under the assumptions of the test. Our results show that all steps must be accounted for when declaring significance in stepwise analytical procedures when a screening step precedes a testing step. A Bonferroni correction for the number of models evaluated could also be applied to a regression after MDR or MDR-PDT, but this would be conservative in the presence of LD.

We also presented a valid test of the null hypothesis of no interaction, and showed it has reasonable performance in scenarios where there are negligible main effects. Our test uses a standard permutation procedure, where disease status labels are exchanged within individuals in the population or sibship without regard to the distribution of genotypes or other covariates. Thus, the null hypothesis of no interaction is not explicitly simulated, although this property does not cause invalid test statistics or lead to large deficits in power. A recent method for searching epistatic spaces with regression, Focused Interaction Testing Framework (FITF) [26], explicitly requires that main effects be present in multilocus models to be detected. The methods here do not have this constraint, and so a broader class of models may be studied.

The power observed in Figure 1 decreased as the MAF increased from 0.2 to 0.4 for all heritability levels; whereas in Figure 2 the opposite trend is observed. This issue seems to be based on the difference between 2 and 3-locus models and the measure of effect size in use here. It is quite difficult to absolutely quantify the strength of the association for purely epistatic interaction models, since there is no baseline or risk allele at any locus. The odds ratio we use here is an improvement over the broad-sense heritability; however, it does seem imperfect for the models in use. Other means of estimating the effect size of purely epistatic models are in development.

Regression in general offers a flexible framework for testing associations between variables. Part of the strength of the regression modeling approach is the specificity with which hypotheses may be tested. However, in the context of modeling interactions from a large space of possible multilocus models, this can also be a weakness. The many possible ways to model interactions, encode genotypes, and correct for multiple comparisons make regression alone cumbersome in epistasis searches. Here, we offer a nonparametric framework for detecting multilocus models, data-driven encoding of genotypes by constructive induction, specifically modeling interactions, and adjusting null distributions of interaction test statistics for the size of the search conducted and the linkage disequilibrium among loci. We show here that an exhaustive analysis using logistic regression with an additive coding is an effective means of detecting purely epistatic 2-locus interaction models; however, this approach is relatively ineffective for 3-locus models. This observation supports the use of MDR-based methods, which were initially designed to search for high-order interactions [6]. Additionally, for the models in this study, conditioning on main effects to detect epistasis would fail, and previous simulation studies have shown this is less powerful than exhaustive searches, even when the interaction models contain main effects [18].

Some future directions of this work will include extending this test of effect modification to more sensitive methods for interaction detection, such as generalized estimating equations (GEE) [27], [28]. This may improve sensitivity in family data [29], and allow the combination of family and case-control data, while preserving specificity, where instead of fitting a logistic model for the best MDR or MDR-PDT model, a GEE model is fit and permutation tested. It is also straightforward to incorporate covariates in the regression models to adjust for potential confounding by population stratification. Additionally, an ordinal outcome framework might be incorporated into the algorithm, allowing for multi-level risk variables [30].

This testing approach is very flexible, and could be adapted to any method searching for epistasis using a permutation test. For instance, one could fit linear regression models for restricted partitioning method (RPM) [31] multilocus models for quantitative traits in the permutation test. RPM is an approach designed to detect purely epistatic models in data with quantitative traits. The approach might also be applied to exotic computational methods such as genetic programming neural networks [32], that use computer learning and evolution principals to search for models. Regardless of the means to search for interactions and the specific test used to evaluate the null hypothesis of no effect modification across genotypes, this framework incorporates the qualities of methods designed to efficiently alleviate the curse of dimensionality and correct for multiple comparisons with the specificity of interaction tests from regression.

## Materials and Methods

### MDR

The MDR procedure has been extensively described elsewhere [2]–[7]. MDR, as shown in Figure S1, is a case-control method for exhaustively searching for and testing multilocus models to detect epistasis. MDR reduces the dimensions of multilocus genotypes to a single binary exposure variable relevant to association by comparing the ratio of cases to controls to a threshold and evaluates models using cross-validation and predictive accuracy. MDR uses K-fold cross-validation to provide some protection against over-fitting results to the data. MDR is model-free and employs a permutation test to determine the significance of a result.

### MDR-PDT

The MDR-PDT is a within-family measure of indirect or direct association between genotype and disease. As described previously [8], the PDT statistic [33] functions within the framework of the MDR algorithm to evaluate multilocus association in pedigrees. MDR-PDT, shown in Figure S2, uses K-fold cross-validation (CV) to manage over-fitting in a way that is analogous to the approach taken by MDR; however, pedigree information is accounted for to achieve an even split of the data for CV [34]. This procedure should mitigate over-fitting by finding models that fit the data well in unobserved samples and avoiding models that fit the training set well but only predict around the expectation of the null [22], [23], [35], [36]. A composite measure of average effect size from the test sets and cross-validation consistency (CVC) is used to rank the models from the search. A within-family permutation test is applied to estimate the significance of the top ranked result, which is adjusted for linkage disequilibrium and the size of the search performed.

### Simulations

The genomeSIMLA [37] software was developed by merging the software packages genomeSIM [38] and SIMLA [39], [40] to simulate pedigree and case-control data with purely epistatic penetrance and more realistic patterns of linkage disequilibrium among the genetic loci. The methods presented here do not assume pure epistasis among loci, meaning interaction models with no marginal main effects, although these models are simulated here to illustrate the capability of this algorithm to detect purely epistatic interactions.

In this study, multiple type I error experiments were performed. The type I error rate of conditional logistic regression with correction for sharing among multiple affected siblings in regions of linkage [41] on MDR-PDT models following an exhaustive search was performed in discordant sibling pair (DSP) datasets (N = 500 DSPs). The type I error rate was determined for logistic regression after an exhaustive search by MDR in datasets with 500 cases and 500 controls. The type I error rates of the regression-based permutation tests for MDR and MDR-PDT were estimated in the same data by comparing the regression likelihood ratio test (LRT) statistic to the critical value from the null distribution of statistics from the permutation test. In each example 1000 replicates were used. Allele frequencies were chosen at random for the simulated non-model SNPs with minor allele frequencies between 0.05 and 0.5 and did not vary across replicates.

In order to provide a comparison of the previously suggested analysis protocol of fitting a regression model of the best MDR or MDR-PDT model to the proposed testing strategy, the best 2-locus model was chosen from a case-control or family-based dataset using MDR or MDR-PDT respectively. Alleles at the 20 loci in these simulations randomly associated and data were simulated for prevalence 0.05 without penetrance parameters for any genotype. Two additional loci were also chosen at random from each dataset as a negative control, and to test the validity of coding the individual SNP genotypes using constructive induction. The three genotypes at each model locus were classified as high or low-risk using MDR or MDR-PDT for both the model loci and the randomly drawn loci. This coding was then used in the regression for each model, and a LRT statistic was calculated for each interaction term and compared to a chi-squared, one degree of freedom distribution for significance. For comparison to conventional analytic techniques for interaction analyses, we also fit logistic regression models with additive encodings for exposure to the minor allele at each SNP, and Bonferroni corrections for the significance threshold after an exhaustive search, as suggested by Marchini et al [17]. For 3-locus interactions, a model including the three marginal effect terms and the three 2-locus interaction terms was fit, evaluating the 3-locus interaction term for significance against a 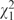 distribution.

Power for the regression extension was measured using purely epistatic models with marginal relative risks <1.001, simulated with a genetic algorithm modified from [42], in genomeSIMLA for 2 and 3 loci, minor allele frequency (MAF) of 0.2 or 0.4, and heritability of 0.03, 0.05 or 0.1. We considered a total of 12 genetic models (Table 2), each of which were simulated as 100, 20-locus datasets for MDR-PDT analysis with independent model loci in 100, 500, 1000 or 2000-DSP pedigrees, or in 100, 50-locus datasets in 200, 500, 1000 or 2000 cases and controls for MDR analysis. Each penetrance table is designed with an odds ratio for exposure to the high-risk genotypes compared to the low-risk genotypes.

**Table 2:** Models examined in the simulation study.

Loci	MAF	Heritability	Odds Ratio
2	0.2	0.030	1.53
2	0.2	0.048	1.79
2	0.2	0.09	3.00
2	0.4	0.03	1.56
2	0.4	0.05	1.79
2	0.4	0.10	2.85
3	0.2	0.03	1.58
3	0.2	0.05	2.10
3	0.2	0.10	3.20
3	0.4	0.03	1.52
3	0.4	0.05	2.23
3	0.4	0.12	3.50

For a set of multilocus genotypes for biallelic loci A and B from a penetrance function F, where pA_i_ (i = 0, 1, 2) is the frequency of the genotypes at locus A, and pB_j_ (j = 0, 1, 2) is the frequency of genotypes at locus B, and i and j count the minor allele at locus A and B respectively. From a penetrance function F, fA_i_B_j_ is the penetrance of the multilocus genotype denoted by indices i and j. Using this notation, the prevalence of the trait K assuming no loss of cases and locus independence is given by Equation 1.
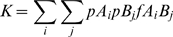
(1)


Also the conditional probabilities of observing a genotype X for a case (Y = 1; Equation 2) or a control (Y = 0; Equation 3) jointly conditional on F can be written.
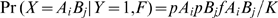
(2)

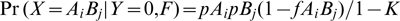
(3)


For a genotype to be considered high-risk for attribute construction using constructive induction by MDR or MDR-PDT in retrospective sampling, the probability of observing the genotype X conditional on Y = 1 must be equal to or larger than the probability of X conditional on Y = 0 (Equation 4).
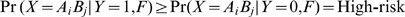
(4)


Otherwise  =  Low-risk

Thereby, using the binary classification of multilocus genotypes as high and low-risk, a 2×2 table relating the exposure to high-risk genotypes to the trait can be constructed (Table 3).
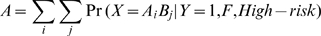
(5)

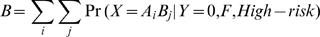
(6)

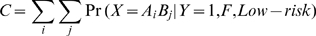
(7)

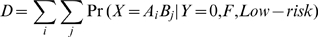
(8)


**Table 3:** Example 2×2 table for the calculation of the purely epistatic disease model odds ratio.

	Case	Control
High-Risk	A	B
Low-Risk	C	D

An odds ratio can be calculated from this table to estimate the effect size for the interaction penetrance model using the standard formula (AD/BC). Since for purely epistatic penetrance models there is no risk allele, and hence no obvious referent (low-risk) genotype, this approach estimates the genotype frequency-averaged odds of disease when exposed to high-risk genotypes divided by the genotype frequency-averaged odds of no disease when exposed to high-risk genotypes.

To evaluate the properties of MDR and MDR-PDT in the presence of independent main effects, pairs of non-interacting loci were simulated in 500 and 2000, 20-marker discordant sibling-pair (DSP) pedigrees and in 500 and 2000 cases and controls. The effect sizes of the model loci were simulated at relative risks 1.5, 2, 4, and 6. The model loci had a dominant model for the minor allele with MAF of 0.2. These data were evaluated for the power of MDR or MDR-PDT to reject the null hypothesis of the permutation test for the 2-locus model featuring the two independent main effects with and without the regression extension.

### Regression Test of Interaction

To conduct a formal test of interaction among the variables in a model resulting from an exhaustive search by MDR or MDR-PDT, the multiple comparisons performed during the search must be accounted for when determining the critical value of the test statistic for significance. Otherwise, when comparing the test statistic to the uncorrected critical value for significance the type I error is inflated. In order to accomplish a valid test of interaction, a straightforward extension to the MDR-PDT and MDR algorithms is implemented.

Where a best two-locus through N-locus model is found by MDR or MDR-PDT, the genotypes at the model loci are determined to be high or low-risk by individual assessment of each model locus by MDR or MDR-PDT. Thereby, for each locus of the N-locus model under consideration, a binary variable is created (X_i_; i = 0, 1, 2, …, N) using the principal of constructive induction and the machinery of MDR or MDR-PDT (Equation 4) which summarizes the marginal risk profile of the genotypes at each SNP. These genotype summary variables are then regressed on the outcome of interest with multiplicative interaction terms (Equation 9).
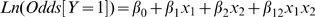
(9)


The regression models for higher-order (3 or more loci) interactions are fit with lower-order interaction terms in the model (Equation 10).
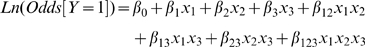
(10)


A regression model is also fit for the reduced model without the highest order interaction term. The negative of twice the difference of the maximized log-likelihoods for these regression models is distributed as 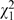 under H_0I_. These regression models are fit with logistic regression (for MDR) or conditional logistic regression models using the method of Siegmund et al [41], implemented in SAS (for MDR-PDT). This method of coding indicator variables reduces the number of interaction terms, and provides a 1 degree of freedom test statistic for any order interaction. Using these regression models, likelihood ratio statistics are calculated for interaction terms to assess effect modification for simultaneous exposure to high-risk genotypes within individuals for each model locus.

To estimate the significance of the multi-locus model the data are permuted as usual, and MDR or MDR-PDT chooses the best two through N-locus model for each permutation. The regressions are fit as in the unpermuted data and the resulting likelihood ratio statistics from each permutation are retained and sorted from largest to smallest. The statistic from the real data is then compared to this distribution to provide an empirical p-value, which is corrected for multiple comparisons (Supplementary Figure S1 and S2).

## Supporting Information

Figure S1 The MDR LR algorithm for evaluating the null hypothesis of no interaction in unrelated cases and controls. Step 1. The data are binned randomly into equal sized bins with the proportion of cases and controls from the full data in each bin for K-fold cross validation. K-1 folds are used in the training set, and the final fold is used as the testing set. The process is repeated K times, so that each bin is used once as a test set. Step 2. For each multilocus genotype, the ratio of cases to controls in the training set is compared to the ratio of cases to controls in the full data. Each genotype with an equal or higher ratio of cases is labeled high-risk, and low-risk otherwise. Step 3. A new binary variable that summarizes risk exposure is constructed by collapsing all cases and controls with high-risk genotypes into one level and all cases and controls with low-risk genotypes into the other level. Step 4. Each model of a given order is evaluated for balanced accuracy (BA) in the training set [11]. BA is defined as the average of the correctly classified cases with high-risk genotypes and correctly classified controls with low-risk genotypes. Step 5. All models of each order (2-locus, 3-locus, etc.) are ranked by BA, with higher values at the top of the distribution. The best model with the highest BA of each order from each training set is recorded. Step 6. The prediction error (PE) in the test set for the best models by BA is calculated using the risk classification established in the training set in Step 3. This value is calculated by dividing the count of cases with low-risk genotypes and controls with high-risk genotypes by the total count of samples in the test set. Step 7. Steps 1–6 are repeated K times so that each cross-validation interval is used one time as a test set. Where the same best model is observed in multiple training sets, a measure of cross-validation consistency (CVC) is observed. A best multilocus model across orders is chosen using the CVC, and then the lowest average PE from test sets as the tiebreaker. Step 8. Full and reduced regression models are fit to the full data using the binary risk classification for genotypes. A single degree of freedom likelihood ratio test statistic is calculated for the highest order interaction term from the regression model. Step 9. A permutation test is conducted by randomly exchanging the status of cases and controls and performing Steps 1–8 at least 1000 times. The observed test statistic from Step 8 is then compared to the ordered distribution of likelihood ratio test statistics to estimate the significance of the result.
(0.66 MB TIF)

Figure S2 The MDR-PDT LR algorithm for evaluating the null hypothesis of no interaction in pedigree data. Step 1. Data are split into k approximately equal parts [25]. Step 2. All possible DSPs and T/UT pairs are generated within each sibship (affected times unaffected) and pooled within k−1/k of the data. This is a training set. Step 3. Each genotype is determined to be high or low risk by comparing the genoPDT statistic [24] from the pooled DSPs and T/UT pairs to a threshold t, such as t = 1, which indicates positive or negative association with affected status. Step 4. Statistics for high-risk genotypes are calculated using the MDR-PDT statistic [8]. Step 5. The procedure repeats for every combination of loci within the order (2-locus, 3-locus, etc.) range specified, calculating an MDRPDT statistic for each, choosing the largest MDR-PDT statistic from each order as the best model at that level. Step 6. The MOR is calculated from the testing set for the best model of each order using the high- low-risk levels established during training. Step 7. Steps 1-6 are repeated in the other splits of the data, so that each CV interval is used as a test set. Where the same model is observed in multiple training sets, a measure of CVC is observed. To select the best from among all models found in training, CVC is considered first, and if necessary the average MOR from test sets can serve as a tiebreaker. Step 8. Full and reduced conditional logistic regression models with the adjustment of Siegmund et al [32] are fit to the full data using the binary risk classification for genotypes from MDR-PDT. A single degree of freedom likelihood ratio test statistic is calculated for the highest order interaction term from the regression model. Step 9. A permutation test is conducted by randomly exchanging the status of cases and controls within sibships and performing Steps 1–8 at least 1000 times. The observed test statistic from Step 8 is then compared to the ordered distribution of likelihood ratio test statistics to estimate the significance of the result.
(0.66 MB TIF)
