# Supplementary figures and images for: A General Framework for Formal Tests of Interaction after Exhaustive Search Methods with Applications to MDR and MDR-PDT
Source: PLoS One. 2010 Feb 23;5(2):e9363. doi: 10.1371/journal.pone.0009363 (PMC2826406; doi:10.1371/journal.pone.0009363)

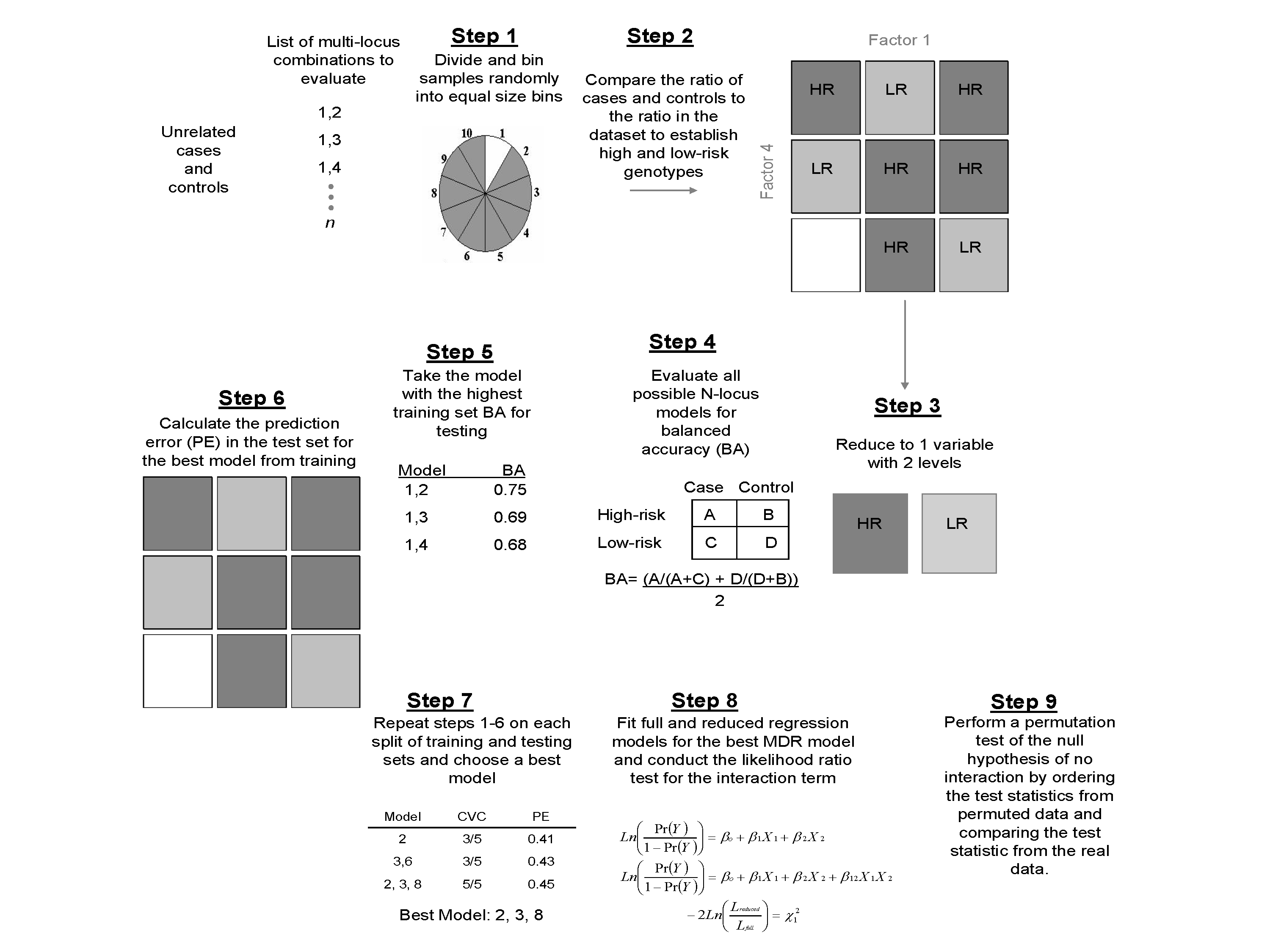

Supplement: Figure S1 — The MDR LR algorithm for evaluating the null hypothesis of no interaction in unrelated cases and controls. Step 1. The data are binned randomly into equal sized bins with the proportion of cases and controls from the full data in each bin for K-fold cross validation. K-1 folds are used in the training set, and the final fold is used as the testing set. The process is repeated K times, so that each bin is used once as a test set. Step 2. For each multilocus genotype, the ratio of cases to controls in the training set is compared to the ratio of cases to controls in the full data. Each genotype with an equal or higher ratio of cases is labeled high-risk, and low-risk otherwise. Step 3. A new binary variable that summarizes risk exposure is constructed by collapsing all cases and controls with high-risk genotypes into one level and all cases and controls with low-risk genotypes into the other level. Step 4. Each model of a given order is evaluated for balanced accuracy (BA) in the training set [11]. BA is defined as the average of the correctly classified cases with high-risk genotypes and correctly classified controls with low-risk genotypes. Step 5. All models of each order (2-locus, 3-locus, etc.) are ranked by BA, with higher values at the top of the distribution. The best model with the highest BA of each order from each training set is recorded. Step 6. The prediction error (PE) in the test set for the best models by BA is calculated using the risk classification established in the training set in Step 3. This value is calculated by dividing the count of cases with low-risk genotypes and controls with high-risk genotypes by the total count of samples in the test set. Step 7. Steps 1–6 are repeated K times so that each cross-validation interval is used one time as a test set. Where the same best model is observed in multiple training sets, a measure of cross-validation consistency (CVC) is observed. A best multilocus model across orders is chosen using the CVC, an [file pone.0009363.s001.tif]

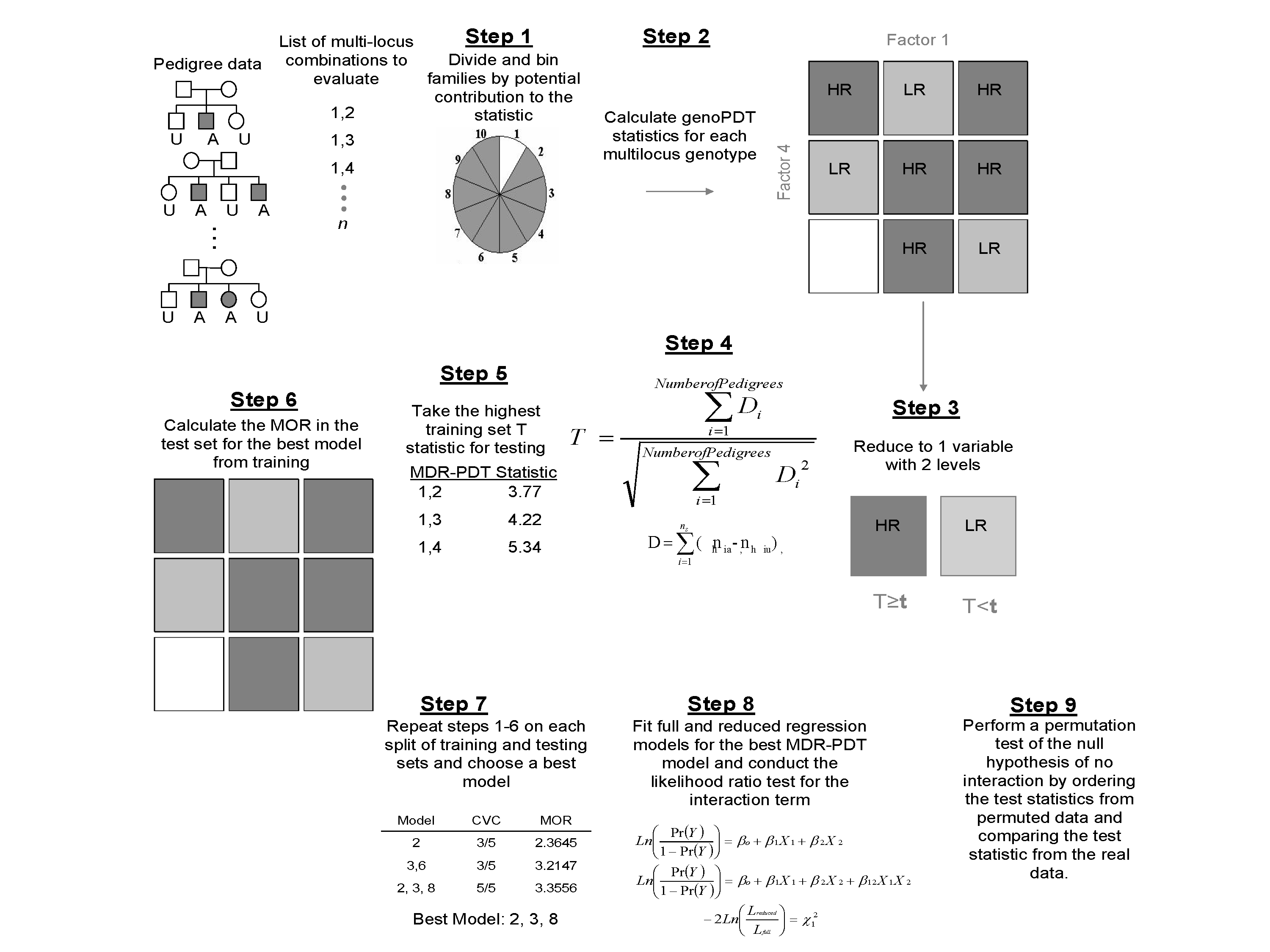

Supplement: Figure S2 — The MDR-PDT LR algorithm for evaluating the null hypothesis of no interaction in pedigree data. Step 1. Data are split into k approximately equal parts [25]. Step 2. All possible DSPs and T/UT pairs are generated within each sibship (affected times unaffected) and pooled within k−1/k of the data. This is a training set. Step 3. Each genotype is determined to be high or low risk by comparing the genoPDT statistic [24] from the pooled DSPs and T/UT pairs to a threshold t, such as t = 1, which indicates positive or negative association with affected status. Step 4. Statistics for high-risk genotypes are calculated using the MDR-PDT statistic [8]. Step 5. The procedure repeats for every combination of loci within the order (2-locus, 3-locus, etc.) range specified, calculating an MDRPDT statistic for each, choosing the largest MDR-PDT statistic from each order as the best model at that level. Step 6. The MOR is calculated from the testing set for the best model of each order using the high- low-risk levels established during training. Step 7. Steps 1-6 are repeated in the other splits of the data, so that each CV interval is used as a test set. Where the same model is observed in multiple training sets, a measure of CVC is observed. To select the best from among all models found in training, CVC is considered first, and if necessary the average MOR from test sets can serve as a tiebreaker. Step 8. Full and reduced conditional logistic regression models with the adjustment of Siegmund et al [32] are fit to the full data using the binary risk classification for genotypes from MDR-PDT. A single degree of freedom likelihood ratio test statistic is calculated for the highest order interaction term from the regression model. Step 9. A permutation test is conducted by randomly exchanging the status of cases and controls within sibships and performing Steps 1–8 at least 1000 times. The observed test statistic from Step 8 is then compared to the ordered distribution of likelihood [file pone.0009363.s002.tif]
